# An upper bound for extreme temperatures over midlatitude land

**DOI:** 10.1073/pnas.2215278120

**Published:** 2023-03-14

**Authors:** Yi Zhang, William R. Boos

**Affiliations:** ^a^Department of Earth and Planetary Science, University of California, Berkeley, CA 94720; ^b^Miller Institute for Basic Research in Science, University of California, Berkeley, CA 94720; ^c^Climate and Ecosystem Sciences Division, Lawrence Berkeley National Laboratory, Berkeley, CA 94720

**Keywords:** heatwave, extreme temperature, convective instability, midlatitude, global warming

## Abstract

Heatwaves damage societies worldwide and are intensifying with global warming. Several mechanistic drivers of heatwaves, such as atmospheric blocking and soil moisture-atmosphere feedback, are well-known for their ability to raise surface air temperature. However, what limits the maximum surface air temperature in heatwaves remains unclear; this became evident during recent Northern Hemisphere heatwaves which achieved temperatures far beyond the upper tail of the observed statistical distribution. Here, we present evidence for the hypothesis that convective instability limits annual maximum surface air temperatures (TXx) over midlatitude land. We provide a theory for the corresponding upper bound of midlatitude temperatures, which accurately describes the observed relationship between temperatures at the surface and in the midtroposphere. We show that known heatwave drivers shift the position of the atmospheric state in the phase space described by the theory, changing its proximity to the upper bound. This theory suggests that the upper bound for midlatitude TXx should increase 1.9 times as fast as 500-hPa temperatures at the time and location of TXx occurrences. Using empirical 500-hPa warming, we project that the upper bound of TXx over Northern Hemisphere midlatitude land (40°N to 65°N) will increase about twice as fast as global mean surface air temperature, and TXx will increase faster than this bound over regions that dry on the hottest days.

## Significance

Heatwaves cause great harm to societies, especially in midlatitude regions that are not adapted to high temperatures. An accurate projection for extremely high temperatures is thus needed to guide adaptation to ongoing global warming. Here, we provide a theory for the upper bound of midlatitude surface temperatures and a scaling for how annual maximum temperatures over midlatitude land will change with global warming.

**Fig. 1.**
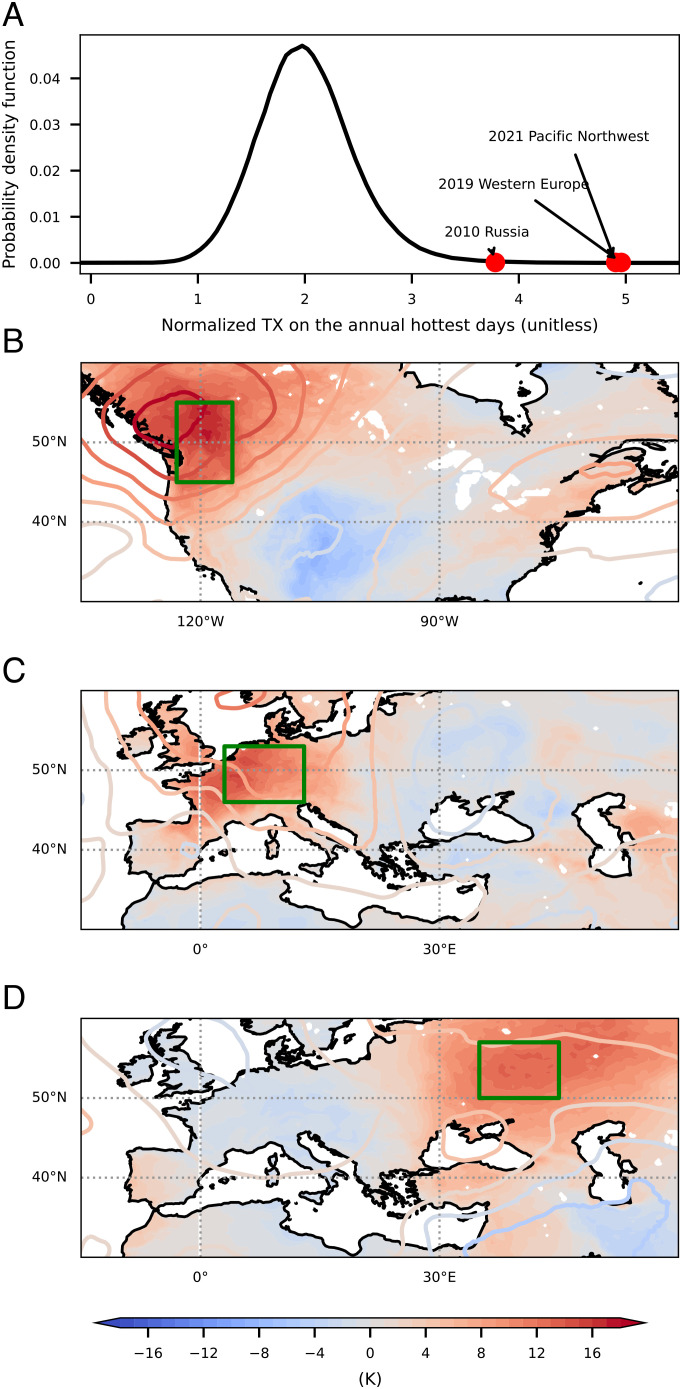
Temperatures of recent mega-heatwaves. (*A*) Probability density of normalized summer (June–August) daily maximum surface air temperatures (TX) on annual hottest days over land between 40°N and 65°N for 1979 to 2021, with maximum TX within affected regions (green boxes in *B*–*D*) during three mega-heatwaves as labeled. Summer TX was normalized by subtracting a daily climatology and then dividing by the SD of summer TX for each location. (*B*–*D*) Surface air temperature anomalies (shading) and 500-hPa temperature anomalies (contours) during three mega-heatwaves: the 2021 Pacific Northwest (*B*), 2019 European (*C*), and 2010 Russian (*D*) heatwaves. Anomalies are relative to a daily climatology, 1979 to 2021. Data use the European Centre for Medium-Range Weather Forecasts Reanalysis 5 (ERA5) at hourly (4), 0.25° × 0.25° resolution.

Recent mega-heatwaves—the 2010 Russian heatwave (1), the 2019 European heatwave (2), and the 2021 Pacific Northwest heatwave (3)—set temperature records more than three standard deviations beyond the local long-term mean of annually hottest daily maximum temperatures (TXx; Fig. 1*A*). The 2010 Russian heatwave (Fig. 1*D*), accompanied by severe drought and wildfires, caused thousands of deaths (5), while the 2019 European heatwave (Fig. 1*C*) exceeded that region’s memorable 2003 heatwave, setting records in Western Europe. The 2021 Pacific Northwest heatwave (Fig. 1*B*), arguably the most anomalous heatwave recorded, exceeded the previous record by 5 °C. Moreover, temperatures in this event broke from the upper tail of the distribution of recorded extreme temperatures, challenging the ability of statistical assessment of its likelihood (3, 6) and calling for a revised physical understanding of heatwaves.

Previous studies identified multiple physical processes involved in midlatitude heatwaves. A prerequisite is an atmospheric anticyclone (7), with clockwise flow (in the Northern Hemisphere) around a high-pressure center. Subsiding air within anticyclones warms through compression, prohibiting clouds and allowing sunlight to heat the surface (8); poleward flow in the anticyclone can also transport hotter air into the heatwave (9). Anticyclones usually drift eastward following midlatitude westerly winds but can stall over a region in a phenomenon known as blocking, which is especially favorable to heatwaves (7). Natural modes of variability that modulate the occurrence and movement of anticyclones thus affect heatwaves (10–14). Beneath anticyclones, land–atmosphere feedbacks can enhance heatwaves (15–17), with warmer air drying soils, which in turn limit surface evaporative cooling and warm surface air more (18–20). Processes that affect soil moisture, such as antecedent precipitation and evapotranspiration (21, 22), therefore affect heatwave severity.

Different heatwaves have been attributed to different processes (23–26), and we lack a general theory for midlatitude heatwave intensity. Furthermore, we do not know whether different processes can interact nonlinearly to amplify heatwaves. This lack of quantitative understanding impedes accurate future projections of extreme temperatures (27). Recent progress, mostly focused on the tropics, suggests that the atmosphere exerts a top–down control on surface air temperature (28–30) and wet-bulb temperature (31–33) through convection. Here, we explore the potential of this perspective for providing a quantitative bound on extreme temperatures over midlatitude land.

## Physical Mechanism and Theory.

We first present a hypothesis, and associated evidence, for the mechanism that limits surface air temperatures over midlatitude land. Specifically, we hypothesize that convective instability halts heatwave development. Surface air temperature cannot increase indefinitely during heatwaves but can only rise until the atmospheric temperature profile becomes unstable to convection, which with any associated precipitation would cool the land surface. This hypothesis requires the free-tropospheric temperature profile to be near neutral to moist convection or, in other words, moist adiabatic, which is an accurate assumption for the tropical atmosphere in general (34, 35). Moist convective neutrality also holds for midlatitude land in summer (36, 37). Here, we study the implication of this moist convective neutrality for midlatitude extreme temperatures and directly demonstrate the role of convective instability in midlatitude heatwaves using observations.

**Fig. 2.**
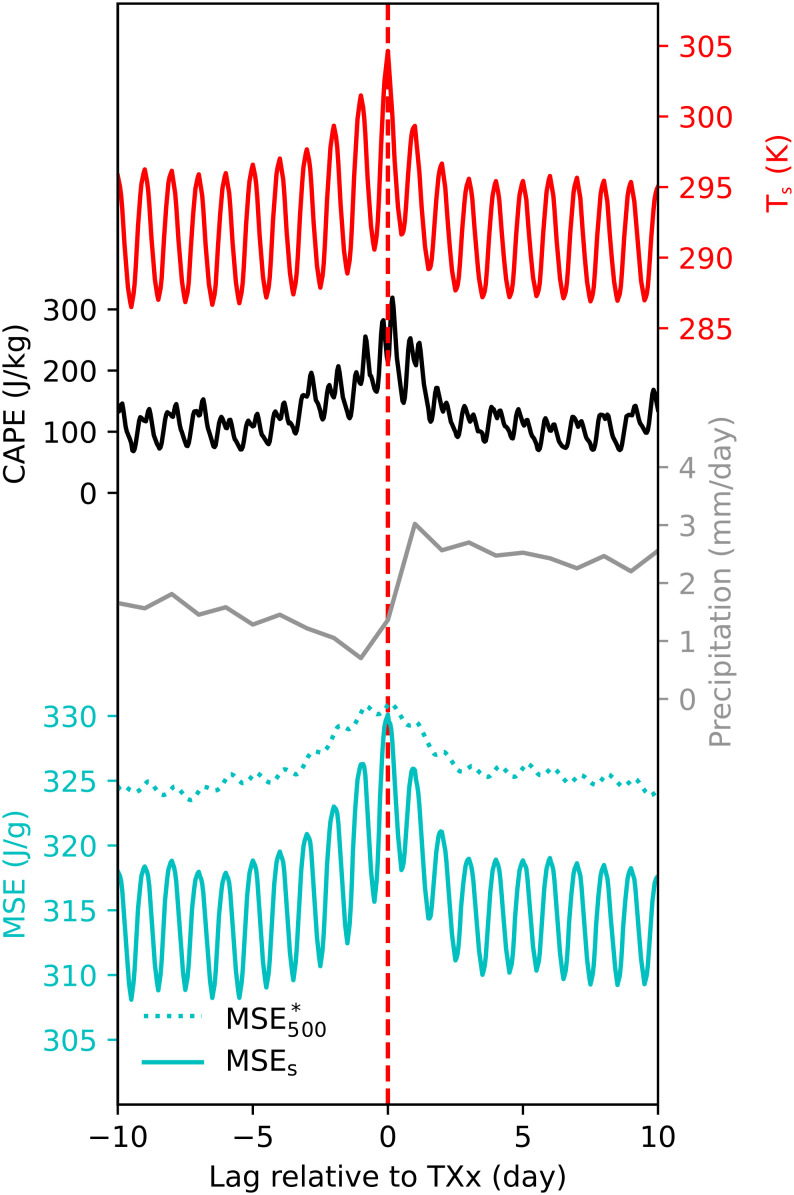
Composite time series centered at annual hottest daily maximum temperatures (TXx). Surface air temperature, convective available potential energy (CAPE), 2-m moist static energy (MSE_surface_), the saturation moist static energy at 500 hPa (MSE_500_^*^) are from hourly reanalysis of ERA5. Precipitation is from GPM daily observations. All time series shown are land averages between 40°N and 65°N of 2010.

We examine this hypothesis using a composite analysis of all annual hottest daily maximum temperatures (TXx) over land between 40°N and 65°N in 2010 (choosing other years does not affect these climatological characteristics). We take the time series of a climate variable over a 21-d window centered on the day of TXx for each location, then average the time series of all locations. The resulting composites (Fig. 2) thus show the structural characteristics of many heat events. Supporting the convective instability hypothesis, convective available potential energy (CAPE), which is a measure of convective instability, peaks on the annual hottest day (day 0). Consequently, precipitation increases on day 0, then surface air temperature drops as precipitation peaks on day 1; this large increase in precipitation over the 3-d period spanning the annual maximum *T*_*s*_ occurs over a large fraction of midlatitude land (*SI Appendix*, Fig. S1). The drop of surface air temperature occurs faster than its build-up, consistent with the hypothesis that the fast processes of convection and precipitation rapidly cool the land surface. These composites identify precipitating convection as a common conclusion of heat events over midlatitude land, motivating application of theories for moist convective stability.

Convective instability can be estimated by comparing surface air moist static energy (MSE) to the free-tropospheric saturation MSE, with the difference between these quantities near zero in the event of convection. MSE depends on temperature (*T*), specific humidity (*q*), and geopotential height (*z*):

[1] $$\begin{array}{c} \mathrm{MSE}=c_{p}T+L_{v}q+gz, \end{array}$$

where *c*_*p*_ is the specific heat of air at constant pressure, *L*_*v*_ is the latent heat of vaporization, and *g* is the gravitational acceleration. Surface air temperature can build in a stable column where surface air MSE (MSE_*s*_) does not exceed free-tropospheric saturation MSE (MSE_a_^*^; calculated by replacing *q* in Eq. **1** with the saturation-specific humidity):

[2] $$\begin{array}{c} {\mathrm{MSE}}_{s}\leq {\mathrm{MSE}}_{a}^{*}. \end{array}$$

Using the 500-hPa level to represent the free troposphere (*Materials and Methods*), we find that midlatitude TXx events satisfy Eq. **2**, with MSE_*s*_ only high enough to reach MSE_500_^*^ on the hottest day (Fig. 2). Combining Eqs. **1** and **2**, and thermodynamic relations, we obtain an upper bound of surface air temperature (*T*_*s*_; *Methods* for derivation):

[3] $$\begin{array}{c} T_{s}\leq T_{500}+\frac{L_{v}}{c_{p}}q_{\mathrm{sat}}\left(T_{500}\right)+\frac{g\bar{z_{500}}}{c_{p}\bar{T_{500}}}T_{500}-\frac{g}{c_{p}}z_{s}, \end{array}$$

where *T*_500_ is 500 hPa temperature, *q*_sat_(*T*_500_) is 500 hPa saturation-specific humidity, $\bar{T_{500}}$ and $\bar{z_{500}}$ are 500 hPa constant climatological values (*Materials and Methods*), and *z*_*s*_ is surface elevation. Eq. **3** states that the highest possible *T*_*s*_ is determined by *T*_500_, offset by *z*_*s*_. The *T*_*s*_ upper bound is achieved when the energy in MSE_*s*_ is entirely allocated to temperature and surface air–specific humidity is zero.

## Observational Evidence.

**Fig. 3.**
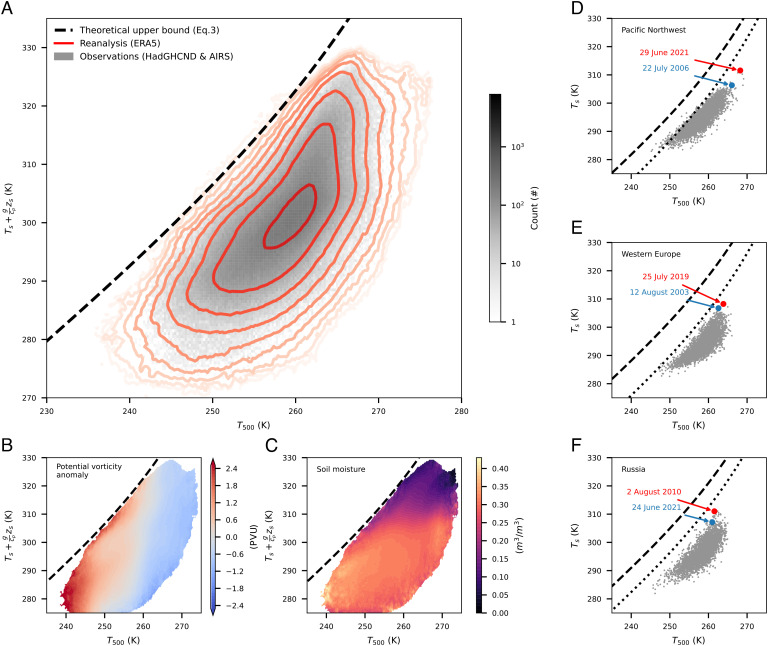
Theory for the upper bound of surface air temperatures, *T*_*s*_, with observational evidence. (*A*) Theoretical upper bound of *T*_*s*_ (black dashed line) and joint histograms of daily-maximum *T*_*s*_ and daily-mean 500-hPa temperatures (*T*_500_) over land between 40°N and 65°N. Gray shading shows combination of *T*_500_ from AIRS (38) and *T*_*s*_ from HadGHCND observations (39) for 2003 to 2014. Red contours (logarithmic scale from 10^2^ to 10^7^) show ERA5 reanalysis for 2001 to 2021. (*B*) Potential vorticity anomaly as a function of *T*_500_ and *T*_*s*_ for 2001 to 2021. (*C*) Same as *B* but for surface-layer (0 to 7 cm) volumetric soil water. (*D*–*F*) *T*_*s*_-*T*_500_ relationship over the green boxed regions in Fig. 1*B*–*D* for 1979 to 2021; dashed lines are the theoretical upper bound and dotted lines are the upper bound minus *L*_*v*_*q*_*s*, min_/*c*_*p*_ where *q*_*s*, min_ is the minimum summer 2-m specific humidity for each region. The hottest day of the most extreme and second most extreme year are marked in red and blue. All panels use data for summer (June–August).

We now assess the consistency of observations with the upper bound expressed by Eq. **3**, examining $T_{s}+\frac{g}{c_{p}}z_{s}$ instead of *T*_*s*_ so that locations with different surface elevations can be readily compared. We show the joint distribution of $T_{s}+\frac{g}{c_{p}}z_{s}$ and *T*_500_ over land between 40°N and 65°N for June, July, and August, with *T*_*s*_ being daily maximum surface temperature and *T*_500_ being daily mean 500-hPa temperature (Fig. 3*A* and *Materials and Methods*). The theory accurately delineates the highest observed $T_{s}+\frac{g}{c_{p}}z_{s}$ for each *T*_500_ (Fig. 3*A*). Few data points fall above the *T*_*s*_ upper bound, where (*T*_*s*_, *T*_500_) pairs would produce convective instability. This analysis only includes the Northern Hemisphere because the same latitudes in the Southern Hemisphere are mostly covered by ocean. The agreement between theory and observations (Fig. 3*A*) suggests *T*_500_ as the limiting factor of *T*_*s*_, providing insight into midlatitude heatwaves.

We argued for a top–down control on *T*_*s*_ by *T*_500_, but causation is not apparent from Fig. 3*A*. To rule out the alternative possibility that *T*_*s*_ controls *T*_500_ through convective heating, we examine the time series of heat events. The 500-hPa saturation MSE (MSE_500_^*^), which strongly depends on *T*_500_, has a broad peak with similar values one day before TXx and on the day of TXx (Fig. 2). If *T*_*s*_ controlled *T*_500_ through convective heating, MSE_500_^*^ would peak after *T*_*s*_ and the onset of precipitation. Instead, the observed cooling of *T*_500_ immediately following the peak *T*_*s*_ indicates that the free troposphere acts to enhance convective instability; this may be associated, e.g., with anticyclones moving out of regions experiencing heat waves, illustrating the relevance of this convective instability limit even when three-dimensional synoptic dynamics operate. Furthermore, individual heatwaves highlighted in Fig. 1*B* –*D* were preceded by warm anomalies confined to the atmospheric layer between 300 and 700 hPa and are succeeded by precipitation (*SI Appendix*, Fig. S2). These time series support the hypothesis that *T*_500_ controls *T*_*s*_ in midlatitude heat extremes, not the other way around.

## Connection to Heatwave Drivers.

We demonstrate how the convective-instability mechanism can be used to understand the influence of anticyclones and soil moisture on heatwaves. We use anomalies (relative to a June–July–August mean) of potential vorticity averaged between 200 hPa and 500 hPa (with an interval of 100 hPa) from reanalysis as a proxy for anticyclone strength, with negative values being anticyclonic in the Northern Hemisphere. As expected, potential vorticity anomalies are anticorrelated with *T*_500_ (Fig. 3*B*), consistent with the expectation that stronger anticyclones are associated with a warmer free troposphere. In the *T*_*s*_-*T*_500_ phase space, anticyclones make warmer *T*_*s*_ possible by moving the atmospheric state to larger *T*_500_. However, the actual *T*_*s*_ achieved in an anticyclone ranges from the upper bound to tens of degrees Celsius below that bound, indicating that strong anticyclones are necessary but insufficient for high *T*_*s*_.

To investigate the role of soil moisture, we examine daily mean volumetric surface (0 to 7 cm) soil water content from reanalysis averaged over the antecedent 30 d. The reanalysis used here, ERA5 (4), assimilates soil moisture observations and represents soil moisture better than previous reanalyses (40). Antecedent surface soil water content at a given *T*_500_ is anticorrelated with *T*_*s*_, with a gradient in *T*_*s*_-*T*_500_ space that is nearly orthogonal to that of anticyclonic strength (Fig. 3*C*). In our convective-instability framework, the role of soil moisture is that drier soil leads to lower surface air–specific humidity (*q*_*s*_) and a partitioning of MSE_*s*_ toward temperature, consistent with the soil moisture–atmosphere feedback (15–20); since the *T*_*s*_ upper bound is only met at zero *q*_*s*_ (*Materials and Methods*), lowering *q*_*s*_ moves the actual *T*_*s*_ toward the upper bound.

To summarize, free-tropospheric anticyclones allow access to larger values of *T*_*s*_ by increasing *T*_500_ (rightward movement in the *T*_*s*_-*T*_500_ phase space), while low antecedent soil moisture allows the actual *T*_*s*_ to approach the upper bound by lowering *q*_*s*_ (upward movement in the phase space). Variations in anticyclone strength and soil moisture align with nearly orthogonal dimensions in the *T*_*s*_-*T*_500_ phase space; neither factor alone ensures a heatwave, while neither factor has to be extreme to result in an extreme heatwave.

## Insight into Recent Heatwaves.

The theory can be applied to the three recent mega-heatwaves in the Pacific Northwest, Western Europe, and Western Russia (Fig. 3*D*–*F*). These regions have moderately humid summers; therefore, the joint *T*_*s*_-*T*_500_ distributions are offset below the upper bound (which assumes zero *q*_*s*_). If we lower the upper bound by the lowest summer *q*_*s*_ achieved over 1979 to 2021 for each region, the maximum *T*_*s*_ then better tracks the adjusted upper bound (Fig. 3*D*–*F*).

Our theory explains the extreme nature of the 2021 Pacific Northwest heatwave, where the highest *T*_*s*_ (29 June 2021) broke the previous record (22 July 2006) by 5 K. For this event, *T*_500_ on 29 June 2021 reached 268.2 K, exceeding the 22 July 2006 value by 2.2 K (Fig. 3*D*), which amounts to a 4.5-K increase in the *T*_*s*_ upper bound by Eq. **3**. Therefore, the *T*_500_ anomaly alone explains most of the 5-K *T*_*s*_ anomaly, and antecedent soil moisture plays a minor role. Our top–down control explanation is consistent with a recent study of this event (41).

For the 2019 Western Europe heatwave (Fig. 3*E*), *T*_500_ on the hottest day (25 July 2019) was 1.3 K higher than the hottest day during the 2003 European heatwave (12 August 2003), translating to a 2.5-K increase in the *T*_*s*_ upper bound. The actual *T*_*s*_ only broke the 2003 record by 1.5 K, consistent with the fact that *q*_*s*_ was higher in the 2019 heatwave. Neither *T*_500_ nor soil moisture broke previous records; *T*_500_ for this event ranked at the top 1.5% and soil water content ranked at the bottom 2% for this region in summer months. This heatwave thus exemplifies the aforementioned near-orthogonal interaction between anticyclone strength and soil moisture in the *T*_*s*_-*T*_500_-pagination phase space.

The 2010 Russian heatwave (Fig. 3*F*) was exacerbated by desiccated soil (*SI Appendix*, Fig. S3*C*) after prolonged blocking. Antecedent soil water content for the hottest days of this heatwave was 36% less than the summer average and 26% less than the summer minimum of other years for the same region, while *T*_500_ only ranked at the 93rd percentile of summer daily *T*_500_ for the region. Compared to the hottest summer day in 2021 (June 24), the excess *T*_500_ on the hottest day in 2010 (August 2) only translates to 2.5 K of increase in the *T*_*s*_ upper bound, but the actual *T*_*s*_ was higher in 2010 by 3.9 K due to desiccated soil; movement in the *T*_*s*_-*T*_500_ phase space was mainly upward relative to the historical distribution.

## Trends of Annual Maximum Temperatures.

We now examine the consistency of historical temperature trends with our theory. The increase of the *T*_*s*_ upper bound (*T*_*s*, max_) per unit warming of *T*_500_ can be obtained by differentiating Eq. **3**:

[4] $$\begin{array}{c} \begin{array}{cc} \frac{dT_{s,\max}}{dT_{500}} & =1+\frac{L_{v}}{c_{p}}\frac{dq_{\mathrm{sat}}\left(T_{500}\right)}{dT_{500}}+\frac{g\bar{z_{500}}}{c_{p}\bar{T_{500}}}.\\ (\mathrm{Magnitude}: & \;+1+0.39∼+1.11+0.21) \end{array} \end{array}$$

Eq. **4** is nonlinear in *T*_500_ due to the near-exponential dependence of *q*_sat_ on temperature, so the sensitivity of *T*_*s*, max_ to *T*_500_ is larger at warmer temperatures. The increase in *T*_*s*, max_ induced by *T*_500_ warming is always larger than the *T*_500_ warming itself, due to contributions from Clausius–Clapeyron (second term on the right hand side of Eq. **4**) and the geopotential (third term). The Clausius–Clapeyron term ranges from 0.39 to 1.11 for *T*_500_ ranging from 250 K and 270 K, which are the 1st and the 99th percentile of *T*_500_ on TXx days over land between 40°N and 65°N. The 500-hPa geopotential anomaly, though frequently analyzed for heatwaves, plays a minor role, contributing about one-fifth that of temperature (first term) and about one-fifth to half that of the Clausius–Clapeyron term. Taking *T*_500_ as 262 K, which is the most common *T*_500_ value on the annual hottest days over midlatitude land in the present climate, we find the increase in the *T*_*s*_ upper bound per unit *T*_500_ warming ($\frac{dT_{s,\max}}{dT_{500}}$) to be 1.86.

**Fig. 4.**
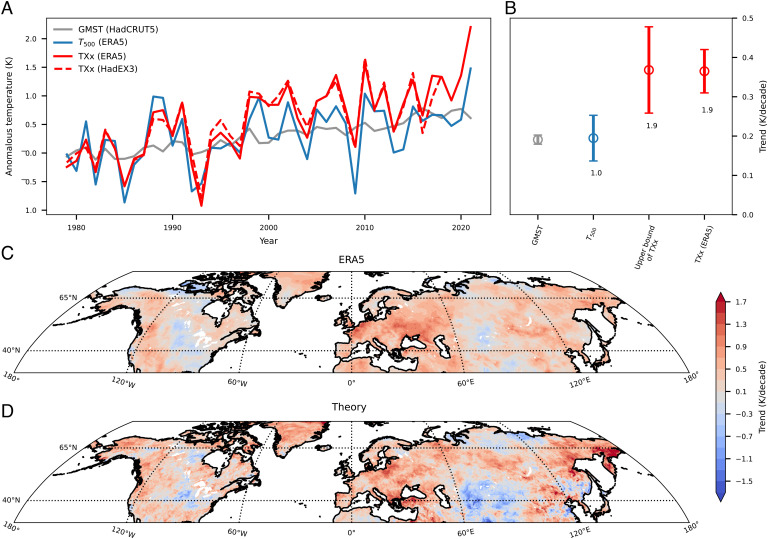
Trends of annual hottest daily maximum temperature (TXx) in agreement with theory. (*A*) Time series of the global mean surface air temperature (GMST) from HadCRUT5 (gray), and the 40°N to 65°N land average of TXx from ERA5 (red solid) and from HadEX3 (red dashed), and *T*_500_ on the annual hottest days from ERA5. (*B*) Trends of GMST, *T*_500_ on annual hottest days, the upper bound of *T*_*s*_, and TXx from ERA5 from 1979 to 2021. Confidence intervals for the linear trends represent 95% significance. Ratios of these trends to the GMST trend over the same period are annotated. (*C*) Location-specific trends of TXx from 1979 to 2021 based on ERA5. (*D*) Same as *C* but for the calculated trends in the upper bound of *T*_*s*_ from theory.

We compare this theoretical ratio with observations and reanalysis (all ranges are 95% confidence intervals of linear trends of annual data points). From 1979 to 2021, the warming of TXx averaged over land between 40°N and 65°N is 1.9 times that of *T*_500_ on such days, from ERA5 reanalysis, with *T*_500_ increasing at 0.19 ± 0.06 K/decade and TXx increasing at 0.36 ± 0.06 K/decade (Fig. 4 *A* and *B*). TXx from HadEX3 (42) gridded station observations increased by 0.32 ± 0.06 K/decade from 1979 to 2018, and *T*_500_ from ERA5 for the same period increased 0.18 ± 0.06 K/decade, with the ratio of the two being 1.8. These similar ratios show that Northern Hemisphere midlatitude TXx increased over recent decades at a rate that agrees strongly with Eq. **4**.

In addition, the spatial pattern of TXx trends resembles that of the *T*_*s*_ upper bound calculated by multiplying the local trend of *T*_500_ on annual hottest days with the local value of $\frac{dT_{s,\max}}{dT_{500}}$ from Eq. **4**. The negative trends of TXx over the Eastern United States and Central Asia correspond to the cooling of *T*_500_ on the hottest days over those regions (Fig. 4 *C* and *D*).

The similar warming trends of TXx and the upper bound of *T*_*s*_ suggest that changes in surface air–specific humidity (*q*_*s*_) on the annual hottest days played a minor role in the land average trend of midlatitude TXx. That is, the long-term movement of the climate state in the *T*_500_-*T*_*s*_ phase space has been nearly parallel to the upper bound (*SI Appendix*, Fig. S5*A*). Drying or moistening of the hottest days should change the proximity of the climate state to the upper bound (*SI Appendix*, Fig. S5*B*) and cause increases in TXx to deviate from the prediction of Eq. **4**. Consistently, the hottest days over most Northern Hemispheric midlatitude land have not seen significant moistening or drying over recent decades (*SI Appendix*, Fig. S4 *A* and *C*), despite the robust increase in annual mean *q*_*s*_ (*SI Appendix*, Fig. S4 *B* and *D*). Though there is uncertainty in *q*_*s*_ data, this result is in line with recent work finding that *q*_*s*_ on the hottest days has a muted increase (43) and has even decreased over certain regions (44).

## Discussion and Implications.

We presented evidence from multiple observational sources supporting the hypothesis that convective instability limits peak surface air temperatures over midlatitude land, and we developed a theory that explains the observed relationship between the peak surface air temperature (*T*_*s*_) and 500-hPa temperature (*T*_500_). This mechanism, focusing on the termination of heatwaves, complements previous descriptions of processes active in the developing phase of heatwaves, providing an upper bound for heatwaves that is a curve in *T*_*s*_-*T*_500_ space.

The direction of causality between *T*_*s*_ and *T*_500_ is important; *T*_500_ warms while convection is suppressed before *T*_*s*_ peaks, then precipitation begins when surface air MSE becomes large enough to satisfy a simple criterion for convective instability (MSE_*s*_ ≥ MSE_a_^*^).

This work seemingly contradicts a common impression that the vertical temperature profile is dry adiabatic (8) rather than moist adiabatic during severe heatwaves, which requires further explanation. The idea that the vertical temperature profile may be dry adiabatic at low levels and moist convectively neutral at upper levels has been used by previous studies investigating the land–ocean mean warming contrast using convective adjustment arguments (28, 45). As shown in *SI Appendix*, Fig. S6, dry adiabatic boundary layers are unusually deep during heatwaves, but temperature profiles in the middle and upper troposphere are still close to moist adiabatic even during the most intense heatwaves. To show that the moist adiabatic part of the troposphere is essential in controlling the maximum surface temperatures, we construct a dry adiabatic equivalent of Eq. **3** by dropping the second term on the right-hand side, i.e., we assume that the 500-hPa level and the surface are connected by a dry adiabat. The dry-adiabatic upper bound underestimates possible *T*_*s*_, and the bias is larger at higher *T*_500_ due to the nonlinearity of the Clausius–Clapeyron relationship (*SI Appendix*, Fig. S7). Furthermore, the dry-adiabatic theory suggests that the *T*_*s*_ upper bound should increase at the same rate as *T*_500_, which does not explain the observed ratio of around 2 in their rates of increase (Fig. 4*B*). A dry-adiabatic theory also cannot explain the observed precipitation jump immediately following peak temperature events (Fig. 2). Therefore, this work recognizes the role of the moist adiabatic free troposphere in limiting *T*_*s*_ and shows that an understanding of extreme *T*_*s*_ focused solely on the dry adiabatic boundary layer is incomplete.

Several caveats exist. First, the simple criterion for convective instability (MSE_*s*_ ≥ MSE_a_^*^) ignores the complexity of temperature and humidity profiles and the entrainment of environmental air into convective plumes (46, 47). In fact, some MSE_*s*_ values do exceed the corresponding MSE_500_^*^. However, this does not disprove the convective instability mechanism, but rather suggests that the theory could be improved if more complex vertical structures of temperature and humidity were considered. Specifically, convective entrainment or convective inhibition (CIN) might explain the rare exceedances of the upper bound visible in Fig. 3*A*; refinement of the theory to account for these factors may thus be warranted. Such refinement may not produce large quantitative changes in our results, as can be illustrated by using CAPE as a measure of the amount of convective instability present if CIN, for example, allowed *T*_*s*_ to increase beyond our current formulation of the upper bound. A dimensionally based scale estimate for the deviation of *T*_*s*_ caused by ignoring CAPE is CAPE/*c*_*p*_; a typical storm-inducing CAPE of 1,000 J/kg would lead to an underestimation of *T*_*s*_ on the order of 1 K.

A second caveat is that, though most locations receive considerable rainfall following heat events, precipitation following heatwaves is much less over dry regions (*SI Appendix*, Fig. S1), such as in Central Asia and the Midwestern United States. The absence of notable precipitation could be due to evaporation of falling condensate, which would not contradict the convective instability mechanism. It could also be due to land–atmosphere interactions that inhibit triggering of moist convection (48, 49), including the presence of convective inhibition (CIN); further investigation of such possible processes is merited.

A natural next step is to estimate how the upper bound of *T*_*s*_ will increase with future global warming. *T*_500_ (from ERA5) on the annual hottest days over Northern Hemispheric midlatitude land has warmed at a similar rate as GMST (HadCRUT5; Fig. 4*B*) in recent decades. Radiosonde and satellite observations of *T*_500_ (50–52) also show similar warming rates as the global mean surface temperature, though the annual mean *T*_500_ is evaluated here due to temporal resolution of data (*SI Appendix*, Table S1). We estimate, using the theory and empirical trends of *T*_500_ and GMST, that the *T*_*s*_ upper bound over midlatitude land should on average increase around twice as fast as GMST. Regional increases of the *T*_*s*_ upper bound depend on the base-state values and warming patterns of *T*_500_. Regions of warmer *T*_500_ in the base climate should expect more increase in the *T*_*s*_ upper bound given the same *T*_500_ warming, due to the Clausius–Clapeyron nonlinearity in Eq. **4**. Further research on the mechanisms, magnitudes, and spatial patterns of *T*_500_ warming with global mean warming is warranted.

A related question is how TXx will change relative to the upper bound of *T*_*s*_, and the answer depends on *q*_*s*_, which depends on multiple processes of land–atmosphere interaction and moisture exchange with neighboring oceans (53). Regions that dry on the hottest days should expect a faster increase in TXx than the upper bound. This work, together with a recent paper on the “drier-get-hotter" mechanism in the tropics (30), emphasizes that trends in extreme temperatures over most of the globe depend crucially on near-surface–specific humidity.

Our results therefore identify two factors that must be constrained for accurate projection of midlatitude extreme temperatures: i) the amount of midlatitude free-tropospheric warming, and ii) surface air–specific humidity changes on the hottest days. Understanding the physical processes controlling these factors should be priority in future research on midlatitude extreme temperatures.

## Materials and Methods

### Derivation of the Upper Bound of Surface Air Temperature.

Combining Eqs. **2** and **1**, we have

[5] $$\begin{array}{c} c_{p}T_{s}+L_{v}q_{s}+gz_{s}\leq c_{p}T_{500}+L_{v}q_{\mathrm{sat}}\left(T_{500}\right)+gz_{500}, \end{array}$$

where *T*_*s*_, *q*_*s*_, and *z*_*s*_ are temperature, specific humidity, and elevation at the surface, respectively,*T*_500_, *q*_sat_(*T*_500_), and *z*_500_ are temperature, saturation-specific humidity, and height at the 500-hPa pressure surface, respectively, *c*_*p*_ of 1004.7090 J/kg/K is the specific heat capacity of air at constant pressure, *L*_*v*_ of 2.5008×10^6^ J/kg is the latent heat of vaporization, and *g* is gravity which equals 9.81 m/s^2^.

We then write *q*_sat_(*T*_500_) and *z*_500_ as functions of *T*_500_, namely

[6] $$\begin{array}{c} q_{\mathrm{sat}}\left(T_{500}\right)≃\frac{\epsilon e_{\mathrm{sat}}\left(T_{500}\right)}{500\;\mathrm{hPa}}, \end{array}$$

where *ϵ* is the molar ratio between water vapor and dry air, *e*_sat_ is the saturation vapor pressure given by the Clausius–Clapeyron equation, and

[7] $$\begin{array}{c} z_{500}=\frac{\bar{z_{500}}}{\bar{T_{500}}}T_{500}, \end{array}$$

where $\bar{z_{500}}$ and $\bar{T_{500}}$ are climatological geopotential height and temperature at 500 hPa, taking the values of 5.682 km and 258.8 K, respectively.

While Eq. **6** is apparent, Eq. **7** requires some elaboration. Combining hydrostatic balance d*p*/d*z* = −*ρg* and the ideal gas law *p* = *ρRT*, we have

[8] $$\begin{array}{c} d\ln p=-\frac{g}{\mathrm{RT}}dz, \end{array}$$

where *p* is pressure, *R* is the ideal gas constant of dry air, with a value of 287.058 J/kg/K. The moist adiabatic lapse rate renders integration of Eq. **8** analytically challenging. Therefore, for this integration only, we approximate the lapse rate as a constant *Γ*, i.e.,

[9] $$\begin{array}{c} T=-\Gamma \left(z-z_{500}\right)+T_{500}. \end{array}$$

This approximation has nothing to do with the assumption of moist neutrality of the atmospheric column at the peak of the heatwave (the central point of this paper), but it allows an approximate expression of *T*_500_ as a function of *z*_500_. We thus can integrate Eq. **8** to get

[10] $$\begin{array}{c} \int d\ln p=\frac{g}{R\Gamma }\int d\ln\left[-\Gamma \left(z-z_{500}\right)+T_{500}\right]. \end{array}$$

We integrate Eq. **10** from the surface (using a nominal value of 1,000 hPa) to 500 hPa, yielding

[11] $$\begin{array}{c} \ln\frac{1000\;\mathrm{hPa}}{500\;\mathrm{hPa}}=\ln2=\frac{g}{R\Gamma }\ln\left(1+\Gamma \frac{z_{500}}{T_{500}}\right). \end{array}$$

The climatological values $\bar{T_{500}}$ and $\bar{z_{500}}$ should also satisfy Eq. **11**:

[12] $$\begin{array}{c} \ln2=\frac{g}{R\Gamma }\ln\left(1+\Gamma \frac{\bar{z_{500}}}{\bar{T_{500}}}\right). \end{array}$$

Eqs. **11** and **12** together give Eq. **7**, which is reasonably accurate for the ERA5 *T*_500_-*z*_500_ relationship.

Substituting Eq. **7** into Eq. **5**, we have

[13] $$\begin{array}{c} c_{p}T_{s}+gz_{s}\leq c_{p}T_{500}+L_{v}q_{\mathrm{sat}}\left(T_{500}\right)+\frac{g\bar{z_{500}}}{\bar{T_{500}}}T_{500}-L_{v}q_{s}. \end{array}$$

We take the maximum of the right hand side of Eq. **13** by setting *q*_*s*_ to zero and thus obtain the upper bound of *T*_*s*_:

[14] $$\begin{array}{c} T_{s}+\frac{g}{c_{p}}z_{s}\leq T_{500}+\frac{L_{v}}{c_{p}}q_{\mathrm{sat}}\left(T_{500}\right)+\frac{g\bar{z_{500}}}{c_{p}\bar{T_{500}}}T_{500}. \end{array}$$

### Choice of the 500-hPa Pressure Level.

The pressure level we choose to represent the free troposphere in the theory should be between the planetary boundary layer (PBL) top and the level of neutral buoyancy (LNB). This level should be far enough from the PBL to not be affected by the surface air temperature, otherwise our theory assuming free-tropospheric control on surface air temperature would not stand; this level should also be frequently coupled to the surface through convection and should be reached by most convective events in summer. The daily-maximum PBL height between 40°N and 65°N on the annual hottest days is around 2 km and could be 5 km over dry areas (based on ERA5), which translates to a PBL top between 550 hPa and 800 hPa. The LNB (calculated from ERA5 hourly data) for summer months between 40°N and 65°N mostly ranges from 250 hPa to 500 hPa. Figures in ref. 36 also show that convective neutrality extends to the midtroposphere for a substantial fraction of time over Northern Hemispheric land in summer. Therefore, we choose the 500-hPa pressure level to represent the free troposphere in Eq. **1**, as it satisfies the two aforementioned requirements.

To test the range of pressure levels where our theory works, we made the same figures as Fig. 3*A* using 400 hPa and 600 hPa temperature. The theory for the upper bound of *T*_*s*_ is modified for new pressure levels accordingly:

[15] $$\begin{array}{c} T_{s,\max}+\frac{g}{c_{p}}z_{s}\leq T_{400}+\frac{L_{v}}{c_{p}}\frac{\epsilon e_{\mathrm{sat}}\left(T_{400}\right)}{400\;\mathrm{hPa}}+\frac{g\bar{z_{400}}}{c_{p}\bar{T_{400}}}T_{400}, \end{array}$$

and

[16] $$\begin{array}{c} T_{s,\max}+\frac{g}{c_{p}}z_{s}\leq T_{600}+\frac{L_{v}}{c_{p}}\frac{\epsilon e_{\mathrm{sat}}\left(T_{600}\right)}{600\;\mathrm{hPa}}+\frac{g\bar{z_{600}}}{c_{p}\bar{T_{600}}}T_{600}. \end{array}$$

Using 400 hPa does not change the result much (*SI Appendix*, Fig. S8*A*), as this level is always above the planetary boundary layer (PBL) and frequently reached by convection. Using 600 hPa results in a deviation from theory at high *T*_*s*_-pagination (*SI Appendix*, Fig. S8*C*); instead of bending upward following the theoretical upper bound, the upper bound of *T*_*s*_-*T*_600_ distribution remains linear for *T*_600_> 310 K. This is because, during these high *T*_*s*_ events, the 600 hPa level is within the boundary layer. *T*_600_ thus cannot be considered as an external limit to *T*_*s*_ and is determined by *T*_*s*_ following a trivial dry adiabat (a linear relationship). This additional analysis supports our choice of 500 hPa and suggests that applying this theory to a pressure level of 600 hPa or lower (in height) is inappropriate.

### Ground Observations.

The HadGHCND dataset provides the anomalies of daily maximum temperatures (TX) on a 2.5 °  × 3.75° spatial grid relative to the 1961 to 1990 climatology. We create a daily TX climatology using ERA5 data interpolated to the coarser grid of HadGHCND.

## Supplementary Material

Appendix 01 (PDF)

## Data Availability

The ERA5 hourly data on pressure levels and single levels from 1979 to present were downloaded from the Copernicus Climate Change Service Climate Data Store (https://cds.climate.copernicus.eu). The 500-hPa temperature data from the Atmospheric Infrared Sounder (AIRS) are downlaoded from NASA Goddard Earth Sciences Data and Information Services Center (https://disc.gsfc.nasa.gov/datasets/AIRS3STD_006/summary). GPM data were downloaded from the NASA Goddard Earth Sciences Data and Information Services Center (https://disc.gsfc.nasa.gov/datasets/GPM_3IMERGDF_06/summary). HadCRUT5 data were provided by Met Office Hadley Centre and downloaded from https://www.metoffice.gov.uk/hadobs/hadcrut5/data/current/download.html. HadEX3 data were provided by Met Office Hadley Centre and downloaded from https://www.metoffice.gov.uk/hadobs/hadex3/. HadGHCND gridded daily temperatures were provided by Met Office Hadley Centre and downloaded from https://www.metoffice.gov.uk/hadobs/hadghcnd/. IUKv2 radiosonde data were provided by Steven Sherwood. MSU/AMSU data produced by Remote Sensing Systems were downloaded from https://www.remss.com/measurements/upper-air-temperature/. All study data are included in the article and/or *SI Appendix*.
