# Supplementary material for: An upper bound for extreme temperatures over midlatitude land
Source: Proc Natl Acad Sci U S A. 2023 Mar 14;120(12):e2215278120. doi: 10.1073/pnas.2215278120 (PMC10041138; doi:10.1073/pnas.2215278120)
Supplement: Supplementary file 1 — Appendix 01 (PDF) [file pnas.2215278120.sapp.pdf]

Supplementary Information

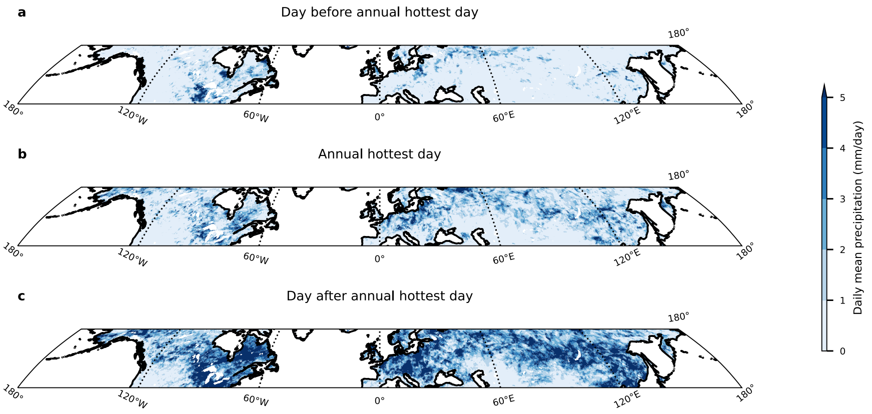

Figure S1: **Location-specific precipitation changes from before to after the annual hottest days.** **a**, daily mean precipitation the day before the annual hottest day averaged over 2010-2020. **b**, same as **a**, but for the annual day. **c**, same as **a**, but for the day after the annual hottest day. Daily mean precipitation observations from GPM are used.

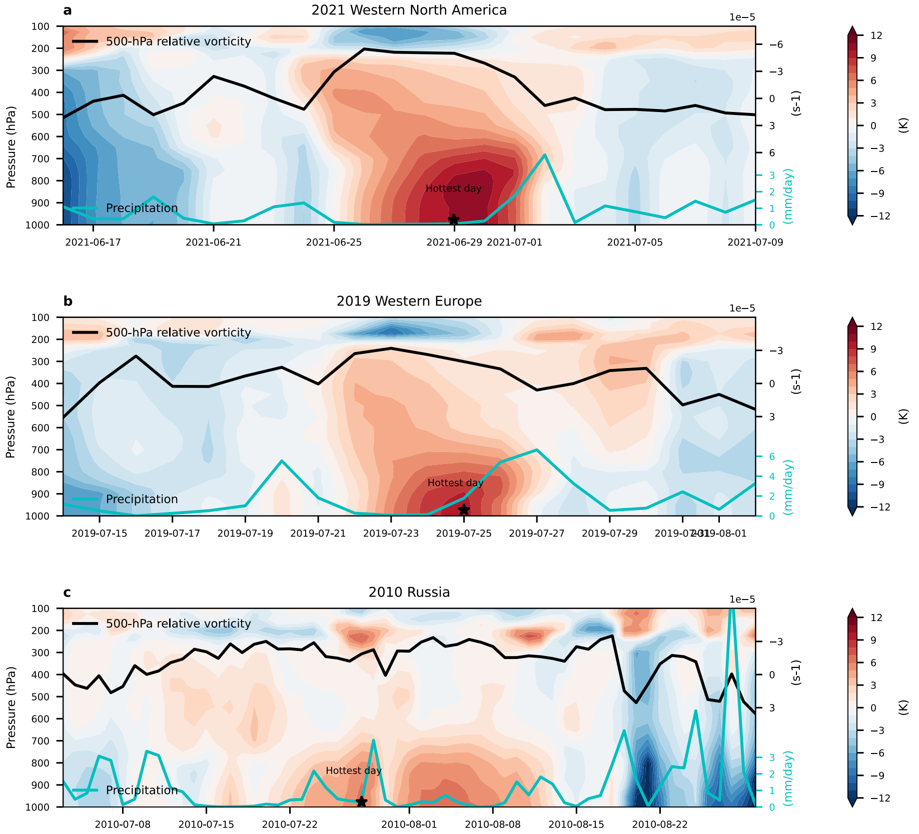

Figure S2: **Time series of three heatwaves**. **a** Daily maximum tropospheric temperature anomalies (color shading), 500-hPa relative vorticity (black line), and precipitation (cyan line) during the 2021 Pacific Northwest heatwave. The same time series are shown for the 2019 Western European heatwave in **b** and the 2010 Russian heatwave in **c**. Average daily-maximum temperature for each vertical level over the shown time periods are subtracted to emphasize the anomalies. Borders of the three regions are the green boxes in Fig. 1b,c,d. Temperature and relative vorticity data are from ERA5, and precipitation data are from GPM.

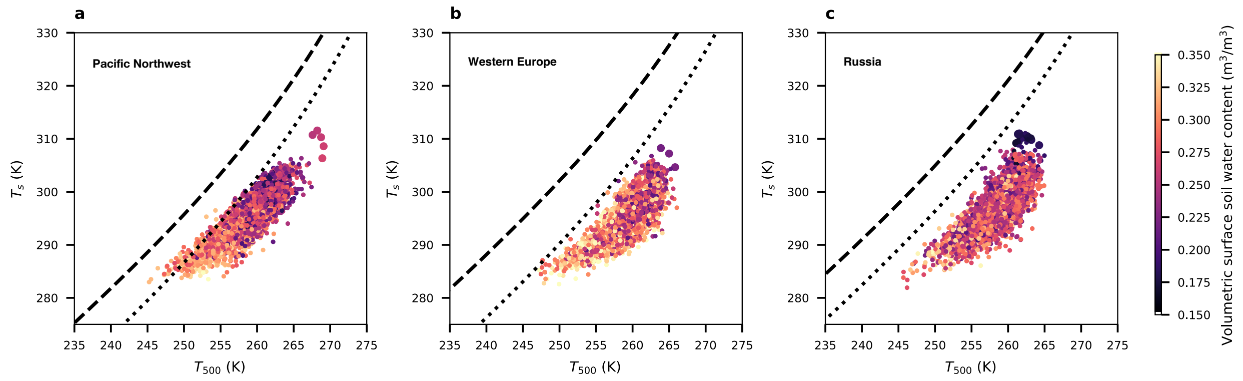

Figure S3: The surface-layer (0-7 mm) volumetric soil water content for June-August from 1979 to 2021 as a function of T_{500} and T_s for (**a**) Pacific Northwest, (**b**) Western Europe, and (**c**) Russia. Dashes lines are the theoretical upper bound of T_s as in equation (3) and dotted lines are the upper bound subtracted by the minimum 2-m specific humidity for these regions.

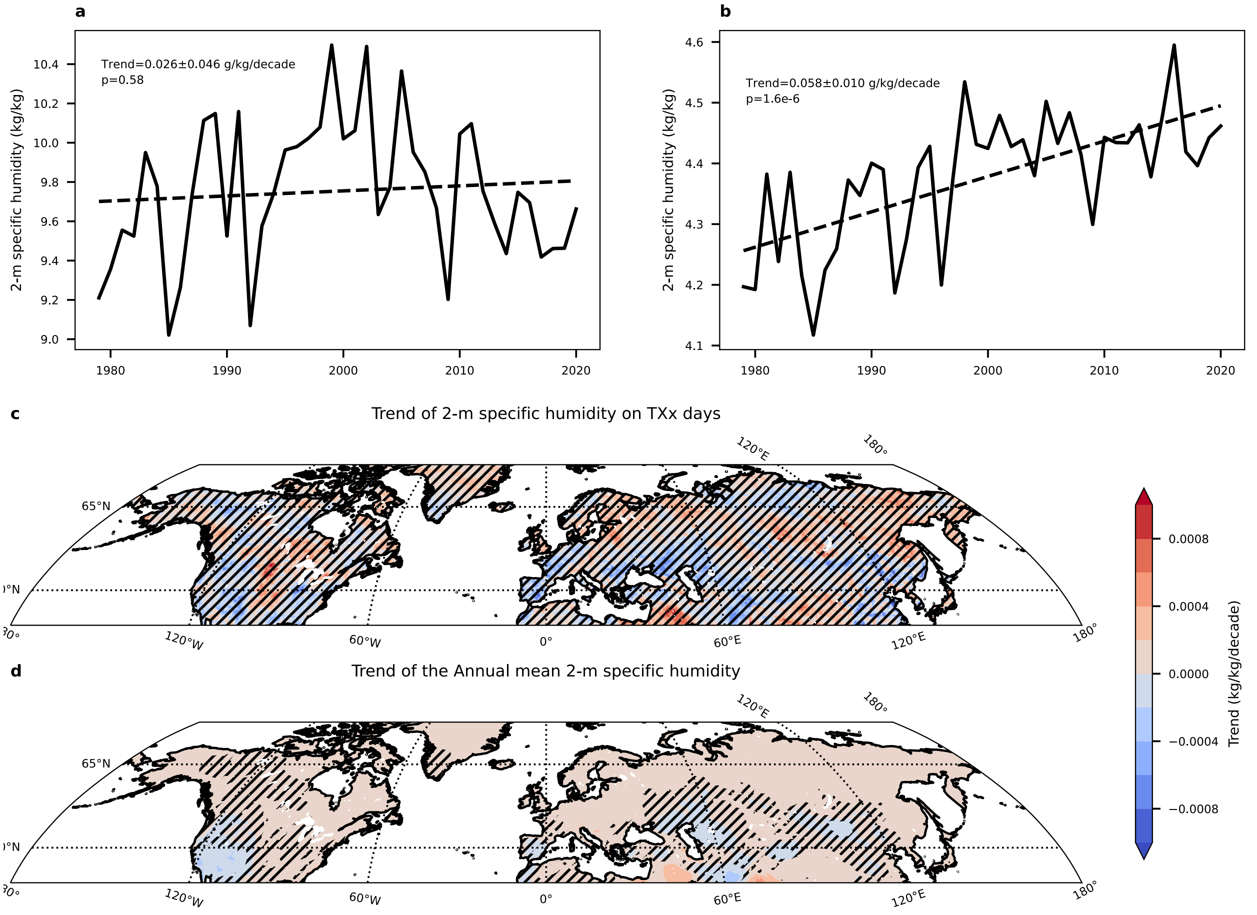

Figure S4: **Trends in the surface (2-meter) air specific humidity.** The time series of **a**, 2-m specific humidity on TXx days and **b**, annual-mean 2-m specific humidity averaged over land between 40°N and 65°N. The location-specific trends of **c**, 2-m specific humidity on TXx days and **d**, annual-mean 2-m specific humidity. Hatched regions are those that did not pass the significant test using a False Discovery Rate (FDR) criterion[54] with $\alpha_{\mathrm{FDR}} = 0.05$.

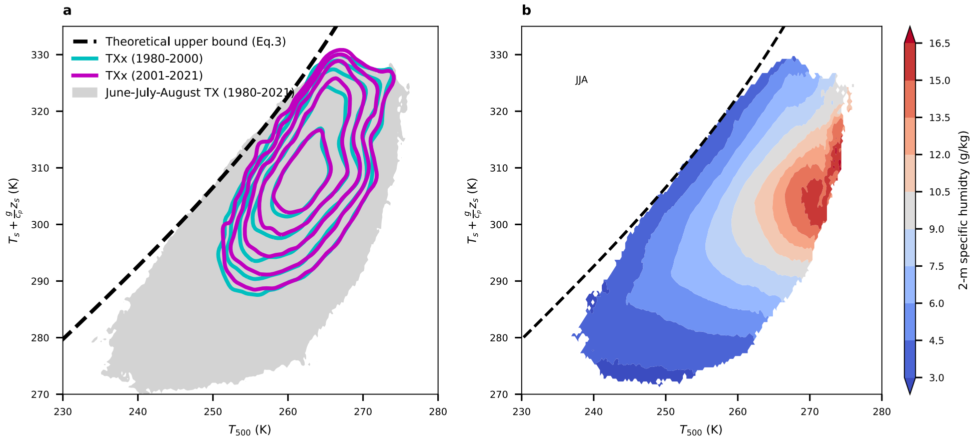

Figure S5: **a**, The T_s-T_{500} joint distributions on the annual hottest days over midlatitude land. The former period (magenta) is from 1980 to 2000 and the latter period (cyan) is from 2001 to 2021. **b**, The 2-m specific humidity as a function of T_s and T_{500}. All data are based on the ERA5 reanalysis.

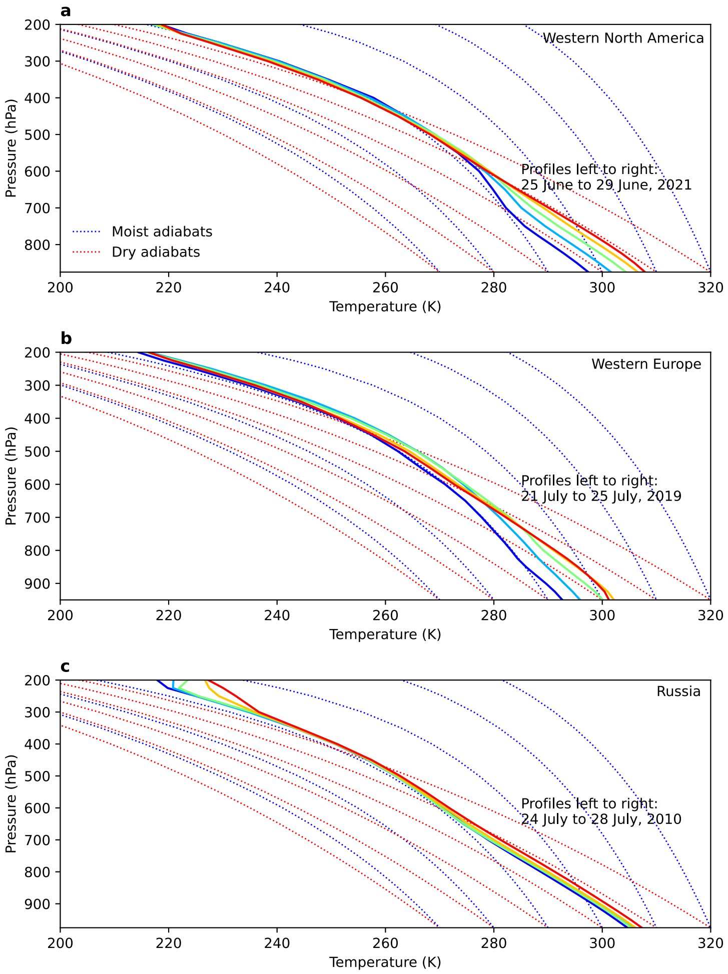

Figure S6: **Free tropospheric temperature is close to moist adiabatic during heatwaves.** **a**, Temperature profiles leading to the hottest days of the 2021 Pacific Northwest heatwave (hottest day 29 June 2021). **b**, the 2019 Western European heatwave (hottest day 25 July 2019). **c**, the 2010 Russian heatwave (hottest day 28 July 2010). Example moist adiabats (blue dotted) and dry adiabats (red dotted) are shown for comparison. Free troposphere is close to moist adiabatic for all three events.

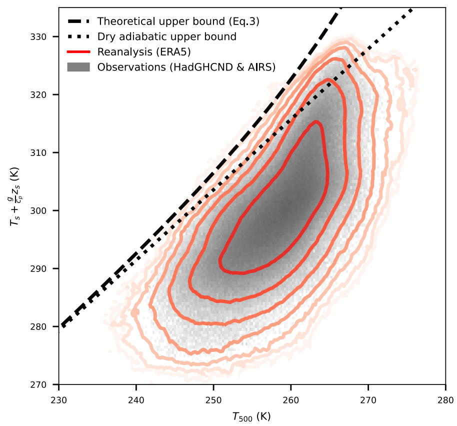

Figure S7: **Dry adiabatic theory does not explain the pattern of T_s and T_{500}.** This is the same figure as Fig. 3a with an additional line indicating the theory based on a dry adiabatic temperature profile between the surface and 500-hPa, which is $T_{s,\max} + \frac{g}{c_p} z_s = T_{500} + \frac{g\overline{z_{500}}}{c_p\overline{T_{500}}} T_{500}$. Theories for $T_{s,\max}$ based on dry and moist adiabats converge at low temperatures.

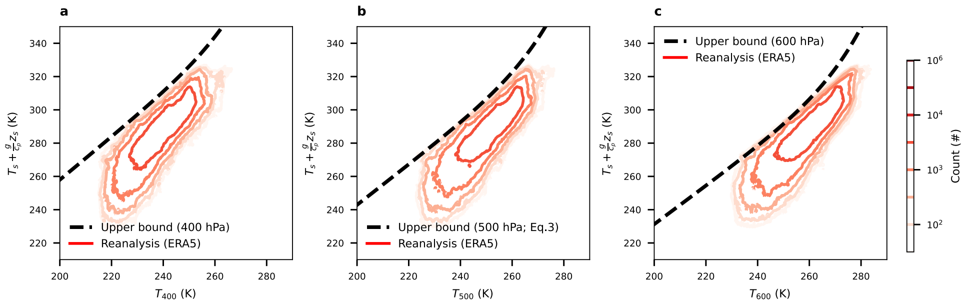

Figure S8: **Theory of the upper bound of T_s using free tropospheric temperature of various levels.** **a**, Theory of the upper bound of T_s (black dashed line) as a function of T_{400} (equation (15)) and joint histogram of daily-maximum temperatures (T_s) and daily-mean 400-hPa temperatures (T_{400}) over land between 40°N and 65°N for 2010 from ERA5. **b**, Same as **a** but for 500 hPa. **c**, Same as **a** but for 600 hPa.

Data set	Time period	Trend normalized by global warming
ERA5 T_{500} on TXx days (land)	1979-2021	1.0 ± 0.3
ERA5 annual mean T_{500} (land&ocean)	1979-2020	1.0 ± 0.2
IUKv2 annual mean T_{500} (land)	1979-2012	1.2 ± 0.4
MSU/AMSU TMT channel (land&ocean)	1979-2020	0.9 ± 0.1
MSU/AMSU TTT channel (land&ocean)	1979-2020	1.1 ± 0.1

Table S1: Average trends of T_{500} between 40°N and 65°N normalized by global warming for multiple data sets. For IUKv2 radiosondes, only the 123 sites with more than 80% of data available during 1979-2012 are included. Error bars are calculated as propagation of uncertainties using the 95% confidence interval of linear trends of T_{500} and global mean surface air temperature assuming the independence of the two variables.
